# How Fiscal Transfers Drive Grain Production: Empirical Evidence from 1319 Counties in China

**DOI:** 10.3390/foods15050820

**Published:** 2026-02-28

**Authors:** Xuezhen Ba, Yu Zhong

**Affiliations:** 1 Party School of Anhui Provincial Committee of C.P.C (Anhui Academy of Governance), Hefei 230022, China; 2 Institute of Agricultural Economics and Development, Chinese Academy of Agricultural Sciences, Beijing 100081, China

**Keywords:** fiscal transfers, grain production, policy effectiveness

## Abstract

Fiscal transfers are a key policy instrument for supporting grain production, and a systematic assessment of their effects offers a critical basis for improving the design of incentive-based grain production policies. Unlike most existing studies, which primarily examine fiscal transfers from the perspective of improving farm households’ welfare and micro-level production decisions, this paper focuses on their impact on the grain production performance of major grain-producing counties, which account for over 80% of China’s grain output. Utilizing panel data from 1319 county-level units in China, this study employs a difference-in-differences (DID) approach to evaluate the impact of the “Reward Policy for Major Grain-Producing Counties (RPMGC)”, a central-to-county fiscal transfer program, on grain production. The empirical results indicate that: First, the reward policy significantly promotes grain production, and this finding remains robust across a series of robustness tests. Second, from a temporal perspective, the policy’s impact follows a trend of initially increasing and then decreasing over time, suggesting that the policy effects lack long-term sustainability. Third, mechanism analysis reveals that the policy enhances grain production by fostering technological advancement, mitigating production risks, and facilitating scaled-up production. Fourth, further analysis indicates that the policy effects are more pronounced in counties located within major grain-producing regions and those experiencing higher fiscal pressure. These findings provide valuable insights for improving the design of intergovernmental grain production incentives, refining grain production incentive mechanisms, and consolidating national food security.

## 1. Introduction

Food security is fundamental to national security and is crucial for managing a wide range of risks and challenges. In China, regional divisions of labor, shaped by variations in natural resource endowments and macroeconomic policies, have concentrated the responsibility for safeguarding food security within major grain-producing regions at the provincial level and major grain-producing counties at the county level. In 2024, the grain output from major grain-producing regions accounted for 77.70% of the national total, representing a 5.03 percentage point increase from 2004. Similarly, the share of output from major grain-producing counties rose from 77.51% in 2005 to 81.77% in 2021, making a substantial contribution to the stable growth of China’s grain production. However, a structural paradox persists: “higher grain production leads to greater economic loss [1,2].” The underlying economic logic is straightforward: as a quasi-public good, food security generates positive externalities. Although the central government has long provided fiscal transfer payments as partial compensation, the opportunity costs associated with grain production, such as foregone non-agricultural land use, environmental pressures, and local fiscal constraints, are difficult to offset fully, resulting in a misalignment between nationally shared benefits and the costs disproportionately borne by major grain-producing counties. In this context, local governments often lack the incentive to actively implement grain production policies. In addition, due to information asymmetry between the central and local governments, abandoning grain production and instead relying on grain circulation to safeguard food security may evolve into a form of “free-riding” behavior, which becomes a dominant strategy for local governments [3]. Furthermore, the long production cycle and tax-exempt nature of grain production limit local governments’ ability to gain promotion opportunities or realize substantial economic returns in the short term [4]. Under a promotion system that prioritizes relative economic performance, local expenditure structures are skewed toward infrastructure and other construction projects that generate rapid short-term growth, resulting in insufficient fiscal support for grain production [5,6].

To address this dilemma, the central government employs fiscal transfer payments as a strategic instrument to realign local incentives. The RPMGC, initiated in 2005, stands as one of the most prominent policy measures. Distinct from general agricultural subsidies, this policy functions as a vertical fiscal compensation mechanism designed to internalize the positive externalities of grain production. Historically, the policy was formally established in April 2005, when the Ministry of Finance issued the “Incentive Measures for Major Grain-Producing Counties.” In terms of policy design, the policy explicitly aims to encourage local governments to prioritize grain production. Eligibility is restricted to county-level units with a five-year average grain output exceeding 200 million kilograms and a commercial grain volume exceeding 5 million kilograms. In terms of funding magnitude, central government allocations have increased steadily, rising from 5.5 billion yuan in 2005 to 57.1 billion yuan in 2024. Theoretically, this substantial infusion of funds should promote grain production by alleviating local fiscal pressures and encouraging investment in agricultural infrastructure. However, critical questions remain: Does this fiscal transfer actually achieve its intended goals? More importantly, how does this policy drive grain production?

Currently, the relevant literature can be broadly grouped into two main strands. The first examines fiscal transfers. As a core policy instrument under fiscal decentralization or federal arrangements, fiscal transfers help strengthen local fiscal capacity and relieve fiscal pressure [7,8]. Empirical studies drawing on evidence from Brazil, Indonesia, Japan, and the United States have linked fiscal transfers to a wide range of outcomes, including environmental protection, economic development, household consumption, corporate tax burdens, and healthcare subsidies [9,10,11,12]. With respect to agricultural production, evidence from Canada, India, and China indicates that fiscal transfers can raise farm household income, ease credit constraints, and stimulate input use. Further research suggests that these income gains are driven largely by higher agricultural output associated with increased input intensity per unit of land [13,14,15]. In addition, studies show that the establishment of China’s “Major Grain-Producing Region” system substantially increased fiscal transfers to designated areas, which, in turn, contributed to higher grain output [16]. The second focuses on the RPMGC. Some studies indicate that the reward policy can enhance grain production by increasing cropping intensity, advancing agricultural mechanization, and promoting technological progress in agricultural machinery [17,18,19]. However, some studies argue that the central government encounters substantial difficulties in effectively monitoring grain production in these counties. Severe information asymmetry leads to only a limited portion of funds being directed toward grain production, which does not significantly enhance overall grain production capacity [20]. Furthermore, Zhang et al. (2020) noted that the policy design may incentivize strategic behavior, causing counties with yields just below the threshold to exaggerate production figures to qualify for incentive funds [21].

In summary, findings regarding the efficacy of the reward policy remain mixed, suggesting that further empirical verification and deeper analysis are warranted. Furthermore, the absence of a publicly available official list of major grain-producing counties makes the identification of a nationally representative sample inherently challenging. Existing studies have often merely examined regional sub-samples or relied on varying screening proxies to identify major grain-producing counties, which may affect the nationwide representativeness and comparability of their conclusions. To address these limitations, this study utilizes a nationwide county-level panel dataset and strictly screens major grain-producing counties in accordance with the eligibility criteria stipulated by the policy, with the aim of comprehensively evaluating how fiscal transfers drive grain production.

The marginal contributions of this paper are fourfold: First, by adopting a rigorous sample identification strategy, this study evaluates the impact of the RPMGC on grain production using a nationwide county-level sample. This provides nationally representative and methodologically robust evidence on policy effects, contributing to a better understanding of the role of fiscal transfers in national food security governance. Second, by introducing a temporal dimension to examine the dynamic evolution of policy effects, this study finds that the policy impact gradually strengthens during the initial implementation phase but subsequently weakens. This provides empirical evidence for discussing the sustainability and the issue of “incentive decay” in such incentive-based fiscal arrangements. Third, we systematically analyze the transmission mechanisms across three dimensions: technological advancement, risk mitigation, and scaled-up production. By delineating how fiscal transfers affect grain production via these channels, we aim to provide a more comprehensive characterization of the policy’s operational pathways than in prior studies. Fourth, we provide detailed evidence regarding heterogeneity. By examining the effects across different major grain-producing regions and varying degrees of fiscal pressure, we offer targeted insights for optimizing the allocation of transfer funds.

## 2. Theoretical Analysis

The positive externalities associated with food security mean that food producers cannot fully capture reasonable returns through market mechanisms alone. As a result, government intervention, particularly fiscal support, is essential to compensate producers for these uncompensated costs [22,23]. In practice, major grain-producing counties bear the important responsibility for safeguarding national food security. They sustain intensive grain production through long-term, resource-intensive investments in land, labor, and capital. Consequently, these counties have been effectively deprived of opportunities to develop secondary and tertiary industries, thereby sacrificing potential development benefits [24]. Moreover, current grain prices fail to reflect the full value of grain production. While production costs have risen rapidly, price increases have lagged, making it difficult for grain production to achieve economy-wide average profits and, in some cases, lead to losses. This means that the more grain major grain-producing counties produce, the higher their opportunity costs and the greater their foregone benefits. Without corresponding compensation mechanisms, it is difficult to maintain the incentives of local governments and farmers to safeguard food security [25]. China’s grain output dropped substantially during 1998 to 2003 and reached 430.7 million tons in 2003, the lowest level recorded since 1990. Against this backdrop, the RPMGC, which is funded primarily through central fiscal transfers, serves as a crucial institutional mechanism for internalizing the positive externalities of food security. Since its introduction in 2005, the policy has played a key role in alleviating fiscal pressures and supporting grain production in major grain-producing counties, with annual central government allocations of approximately 30 billion yuan.

The incentive funds are linked to factors such as grain output, sown area, and performance evaluations, and are allocated as general transfer payments that are managed and utilized autonomously by county-level governments. This design aligns the urgently needed fiscal support for county governments with grain production performance within their jurisdictions. Moreover, the flexibility and autonomy in fund allocation align with county governments’ needs in areas such as fiscal security, grain production, and public service provision, thereby incentivizing local governments to increase grain production in order to maintain eligibility for the incentive funds. Simultaneously, the performance evaluation mechanism further reinforces the central government’s accountability as the principal overseeing fund utilization and enhances the accountability of county governments as agents responsible for safeguarding food security. For example, Jiangxi stipulates in its evaluation system that “20% of the reward funds for major grain-producing counties should be allocated to grain production and industry development,” whereas Chongqing requires that “over 50% of major grain-producing counties’ funds be used for grain and oil production.” These measures, which restrict the permitted use of a portion of the funds, help county governments increase fiscal investments in grain production and more effectively achieve the grain-increasing effects of the RPMGC.

Furthermore, within the frameworks of fiscal federalism and principal–agent theory, the RPMGC can be interpreted as a performance-based fiscal transfer through which the central government, as the principal, strengthens the incentives of county governments, as agents, to prioritize grain production. In addition to providing fiscal resources, the policy also conveys symbolic recognition: being designated and rewarded as a major grain-producing county sends a positive political signal about local performance in safeguarding food security. This combination of fiscal and political incentives encourages county governments to devote greater attention to grain-related responsibilities and to allocate more budgetary and administrative resources to grain production. It is noteworthy, however, that previous studies indicate a gradual decline in the contribution of reward funds for major grain-producing counties to grain output, falling from 0.09 tons per 10,000 yuan in 2005 to 0.02 tons per 10,000 yuan in 2017 [26]. Other studies similarly suggest that the slowing growth rate of reward funds in recent years has weakened their grain-increasing effects [18,27]. Accordingly, we propose Hypothesis 1.

**Hypothesis** **1:** *The RPMGC promotes grain production, but its effect may diminish over time.*

**Figure 1:**
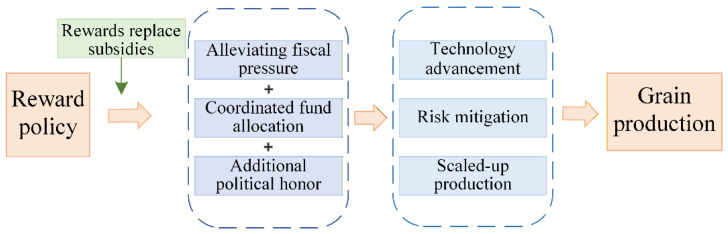
Analytical Framework.

The fundamental mechanism through which the RPMGC promotes grain production at the county level is as follows: The central government alleviates fiscal pressure on county governments by providing incentive-based fiscal transfers, while simultaneously encouraging increased investment in grain production through flexible fund allocation, targeted policy guidance, and the political recognition associated with receiving these funds (Figure 1). Based on a review of the specific uses of incentive funds, the policy primarily supports stable and sustained increases grain production through three mechanisms: fostering technological advancement, mitigating production risks, and facilitating scaled-up production.

From the perspective of fostering technological advancement, agricultural technological progress primarily takes two forms: mechanization, which substitutes for labor, and biotechnological and biochemical technologies, which substitute for land. These technologies are typically introduced into production processes through capital investment, incorporating technological improvements into grain production via agricultural machinery, fertilizers, and pesticides [28]. Under the incentives of the RPMGC, county governments have increased fiscal investment in areas such as subsidies for agricultural machinery purchases, mechanized agricultural services, and fertilizer and pesticide use. The resulting private investment substitution effects and indirect income effects further enable grain farmers to allocate more production factors, including machinery and fertilizers. The significant contributions of agricultural mechanization and fertilizer inputs to grain yield per unit area have been widely documented in the literature [29,30].

From the perspective of mitigating production risks, grain production is a process that combines natural reproduction with economic reproduction. This inherent vulnerability makes it highly susceptible to natural hazards. Studies have shown that natural hazards can reduce grain yields by approximately 5–10% [31], and areas affected by droughts and floods account for the largest share of climate-related hazard-affected areas [32]. A persistent tension remains between the uncertainty associated with climate change and the objective demand for stable and high grain yields. The RPMGC incentivizes county governments to implement projects such as farmland water conservancy and high-standard farmland construction, which address crop irrigation needs and improve irrigation efficiency. These measures reduce reliance on weather conditions, enhance risk resistance and resilience, thereby ensure stable grain output and promote increases in grain yield per unit area.

From the perspective of facilitating scaled-up production, with the large-scale migration of rural labor, scaled-up production has become an objective necessity and an inevitable trend in the modernization of Chinese agriculture. Theoretically, moderate scaled-up production can enhance the specialization in grain production by optimizing resource allocation, improving market-based allocation of production factors, and promoting the adoption of advanced technologies [33]. Numerous studies also demonstrate that grain yield per unit area increases with the expansion of production scale and declines only when the scale exceeds an optimal range [34]. Considering China’s reality as a “large country with small farmers,” operations are likely still within this optimal scale range. The RPMGC provides fiscal support to county governments to facilitate appropriately scaled production, potentially allowing counties to improve the deployment of key inputs, including land, capital, and labor, and thereby increase grain yield per unit area. Accordingly, we propose Hypothesis 2.

**Hypothesis** **2:** *The RPMGC promotes grain production through the pathways of fostering technological advancement, mitigating production risks, and facilitating scaled-up production.*

## 3. Research Design

### 3.1. Sample Description

To evaluate the impact of the RPMGC on grain production, this study constructs annual panel data for Chinese counties from 2000 to 2021. To ensure sample representativeness and to minimize potential selection bias arising from regional heterogeneity, we applied the following rigorous data-processing procedures. First, given the limitations in the reward policy’s coverage and data quality, the sample excludes Beijing, Tianjin, Shanghai, Tibet, Hong Kong, Macao, and Taiwan. These regions are highly urbanized, with relatively weak grain production functions, and some are not direct targets of the policy. Therefore, their exclusion is unlikely to cause systematic bias in identifying policy effects for typical major grain-producing counties. Second, municipal districts, special districts, and forest areas are excluded because they are not comparable to general county-level units in terms of grain production and economic indicators. Third, using China’s 2021 administrative boundaries as the benchmark, we exclude county-level units that experienced boundary changes during the sample period. This prevents misclassification of treatment status caused by boundary changes and variations in statistical criteria for outcome variables such as grain output, thereby reducing the influence of measurement errors on policy effect estimation. Fourth, after further excluding counties with substantial missing data, the final sample comprises 1319 county-level units. Missing observations are filled in using linear interpolation, outliers are winsorized, and all monetary measures are deflated to 2000 prices with the provincial CPI.

Data sources include the China County Statistical Yearbook, the CSMAR database, the China Statistical Yearbook, the county-level crop database of the China Crop Production Information Network, GSOD meteorological station data released by the U.S. National Centers for Environmental Information, and historical statistical archives/local records from provincial, municipal, and county authorities.

### 3.2. Variable Selection and Data Sources

Explained variable: Grain yield per unit area (Yield). Given the limited scope for expanding arable land in China, this indicator serves as a key measure of grain production capacity [18].

Explanatory variable: RPMGC (Policy). The treatment effect is identified by the estimated coefficient on post × treat, where treat indicates major grain-producing counties and post denotes the post-policy period. According to the eligibility criteria specified in the Ministry of Finance’s 2005 Central Government Incentive Measures for Major Grain-Producing Counties, which require a five-year average grain output exceeding 200 million kilograms and a commercial grain output exceeding 5 million kilograms, 498 counties continuously receiving the reward and 821 counties never receiving it are identified.

Variables for mechanism analysis: Based on theoretical analysis and the extant literature [35,36,37,38], we posit that the reward policy operates through three channels: technological progress, production risk mitigation, and scaled-up production. Accordingly, four mediating variables are selected as proxies: agricultural mechanization level, fertilizer application amount, effective irrigated area, and cultivated area per laborer. (i) Technological progress is measured by agricultural mechanization level and fertilizer application amount. Agricultural mechanization reflects innovations in grain production tools, substitutes for agricultural human capital, and represents major changes in production methods with substantial gains in productivity. Fertilizer application amount captures advancements in biochemical technology, serves as a substitute for land inputs, and is a key technology for improving land productivity. (ii) Production risk mitigation is measured by effective irrigated area. Expanding this area mitigates weather-related risks and improves farmland drought resilience, which is increasingly important in the context of more frequent climate-induced natural disasters. (iii) Scaled-up production is measured by cultivated area per laborer. In agricultural economics, cultivated area per laborer is widely used as a proxy for operational scale [39,40], as it reflects the extent to which land is consolidated into larger production units, given the local labor endowment.

Control variables: Consistent with previous studies [17,41], we include control variables covering macroeconomic and natural environmental factors that may influence grain production. Macroeconomic variables include the share of the primary industry, the share of the secondary industry, government size, level of financial development, household savings, and regional area. Natural environmental variables include average annual temperature, annual precipitation, and crop-sown area (as shown in Table 1).

### 3.3. Model Specification

Following Wang et al. (2024) [42], we use DID design to identify the effect of RPMGC on grain production. The baseline specification is written as follows:

(1) $$y_{ct}=\alpha +\beta post_{t}\times treat_{c}+\sigma x_{c}+\mu_{c}+\gamma_{t}+\varepsilon_{ct}$$

In the specification, $y_{ct}$ is the explained variable. $post_{t}\times treat_{c}$ is the explanatory variable. The coefficient of interest is β, where a significantly positive estimate implies that RPMGC increases grain production. $x_{c}$ denotes a vector of controls. $\mu_{c}$ and $\gamma_{t}$ are county and year fixed effects. $\varepsilon_{ct}$ is the error term. Standard errors are clustered at the county level.

## 4. Empirical Findings

### 4.1. Benchmark Analysis

Table 2 reports baseline results. In Column (1), we estimate a model with county and year fixed effects only. The RPMGC coefficient is 0.298 and statistically significant at the 1% level, indicating that the program is linked to higher grain yield per unit area. Column (2) adds a vector of controls; the estimated policy effect increases to 0.316 and remains statistically significant at the 1% level, leaving the main finding unchanged. These results suggest that across different identification strategies, the estimated coefficients remain positive and highly significant, indicating that the implementation of the RPMGC substantially enhances grain production. Based on the estimates in Column (2), the RPMGC increases grain yield per unit area by approximately 0.316 tons relative to the control group. This finding is broadly consistent with the results of Luo et al. (2024) [18], who report that the RPMGC increases grain yield per unit area by 0.31 tons, thereby further confirming the robustness of the empirical results. This outcome indicates that, under increasingly stringent constraints on China’s arable land resources and the diminishing potential for expanding grain production through enlarging planting areas, the RPMGC, by leveraging fiscal incentives, effectively focuses on unlocking and enhancing grain yield per unit area. Accordingly, it offers a feasible policy pathway for strengthening grain supply capacity within the constraints of existing land endowments.

### 4.2. Parallel Trend Test

A prerequisite for policy evaluation using DID framework is that the treatment and control groups exhibit the same pre-policy trend. In other words, prior to the implementation of RPMGC, there should be no statistically significant difference in grain yield per unit area between the matched treatment sample and the matched control sample. Accordingly, this study adopts an event-study specification to test the parallel trends assumption and to examine the dynamic effects of RPMGC.

**Figure 2:**
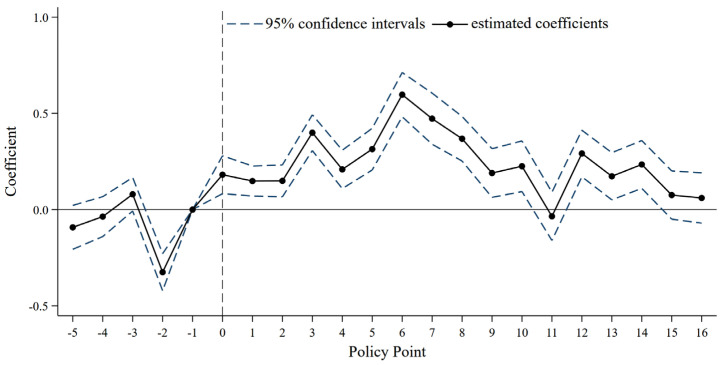
Parallel trend test results.

Figure 2 shows that the treatment and control groups follow similar pre-trend patterns, with no clear divergence in yields. The year 2003, however, is an outlier: China’s grain output declined sharply by 5.8% compared with 2002, and major grain-producing counties, the primary bases of grain production, were disproportionately affected. According to the literature, several regions in China experienced severe natural and climatic shocks, including spring drought, low-temperature frost, flooding, heat stress, and multiple pest and disease outbreaks in 2003. Nationwide, the affected area, disaster-stricken area, and total crop-failure area reached 54,506, 32,516, and 8546 thousand hectares, respectively, representing increases of 7387, 5197, and 1987 thousand hectares relative to 2002. In addition, reductions in grain-sown area due to the Grain-for-Forest Program and crop-structure adjustments aimed at reducing grain cultivation further exacerbated the decline in grain yield per unit area in 2003. Following the implementation of the RPMGC, the estimated policy coefficient remains consistently positive and shows an initial increase followed by a gradual decline over time.

Specifically, the policy’s beneficial effect on grain yield per unit area was already evident in the year of its implementation. After peaking in 2011, its effectiveness gradually diminished, and the policy’s impact had declined substantially by 2016, 2020, and 2021. The policy delivered significant early gains, which appear closely associated with enhanced fiscal incentives and the rollout of a performance evaluation mechanism. This pattern supports the argument that stronger incentives at the outset can generate a larger response. The subsequent decline in the policy’s effectiveness is likely related to the slowing growth rate of incentive funds for these counties. Data shows that incentive funds grew at an average annual rate of 25.24% prior to 2014. After 2014, the growth rate fell below 10%, reaching 5.87% in 2016 and declining further to 3.78% in 2020 and 3.70% in 2021. The timing of this slowdown is consistent with the observed weakening of the policy effect, it is therefore reasonable to conjecture that the reduced growth of fiscal incentives may have contributed to the decline in policy effectiveness. In summary, the RPMGC has substantially enhanced county-level grain production, but its effectiveness has gradually diminished over time, thereby providing empirical support for Hypothesis 1.

### 4.3. Robustness Tests

**Figure 3:**
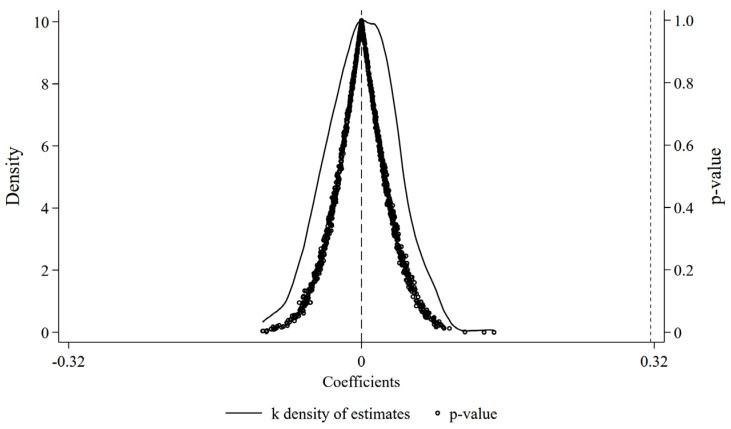
Placebo test results.

Placebo Test: To examine whether the estimated RPMGC effect could be contaminated by unobserved confounders (such as omitted factors), we conduct a randomization-based placebo analysis. Specifically, a random list of major grain-producing counties was drawn from the full sample, matching the actual number of major grain-producing counties, and a fictitious policy implementation date was randomly assigned. Model (1) was subsequently re-estimated 1000 times, and the resulting estimates were recorded to enhance the re-liability of the test. Figure 3 summarizes the simulation outcomes: placebo coefficients cluster around zero and most associated *p*-values exceed 0.1, while the benchmark estimate of 0.316 reported in Table 2 falls in the far-right tail of the placebo distribution. Overall, the placebo results suggest that the baseline finding is unlikely to be generated by random assignment or unaccounted shocks, supporting the robustness of the benchmark estimates.

Instrumental Variables Method: This study employs the instrumental variable (IV) approach to address potential endogeneity concerns, providing an additional robustness check for the benchmark regression results. Specifically, farmers’ “three-grain reserves” (subsistence, feed, and seed grain) are selected as instrumental variables. According to the calculation rules stipulated in the RPMGC, subsistence grain is defined as per capita rural grain consumption, and feed and seed grain are set at 175 kg in southern regions and 225 kg in northern regions. Population size is measured by the number of rural residents.

A valid instrumental variable must meet two requirements, namely, relevance to the endogenous variable and exogeneity. The rationale for selecting this IV is as follows. First, the size of farmers’ “three-grain reserves” significantly affects each county’s commercial grain output. In other words, lower levels of farmers’ “three-grain reserves” are associated with a higher likelihood of receiving rewards, which satisfies the relevance condition for the instrument. Second, farmers’ “three-grain reserves” are calculated based on local grain consumption habits, crop and livestock production structure, and population size, while the quantities of feed grain and seed grain are determined according to fixed policy parameters, such as region-specific standards for northern and southern China. These factors are unlikely to be directly related to the dependent variable, thereby supporting the exogeneity assumption of the instrumental variable. As shown in Table 3, the estimated coefficient in the first stage is negative, indicating that higher levels of farmers’ “three-grain reserves” reduce the likelihood that a county receives incentives under the RPMGC. The second-stage coefficient is positive, suggesting that after addressing endogeneity concerns, the policy continues to exert a positive effect on grain yield per unit area. In addition, the regression results indicate that the IV passes both the Kleibergen–Paap rk LM test for under-identification and the Kleibergen–Paap rk Wald F test for weak identification. These results further confirm that using farmers’ “three-grain reserves” as the IV is robust and appropriate.

Other Robustness Checks: This study conducts a series of additional robustness checks, and the regression results are summarized in Table 4.

Replacement of the Explained Variable: In the benchmark regression, grain yield per unit area serves as the main indicator of county-level grain production capacity. However, total grain output and sown area are also important measures of production performance. To test the robustness of the benchmark results, Model (1) is re-estimated using the natural logarithms of total grain output and sown area as the dependent variables. The results are presented in Columns (1) and (2) of Table 4. We obtain positive policy estimates of 0.231 and 0.206, both significant at the 1% level, providing further support for the robustness of the preceding findings.

Adjustment of Sample Size: Column (3) drops the 2005 observations and re-estimates the model. The policy estimate equals 0.308 and is still statistically significant at the 1% level. Given that the provincial governor responsibility system for food security may introduce additional policy shocks, Column (4) further excludes the 2015–2021 subsample. The RPMGC estimate stays positive and statistically significant, suggesting that the main conclusion is robust to these sample restrictions.

Adjustment of Control Variables: Considering that the control variables may also be influenced by the RPMGC, following Li et al. (2016) [43], the interaction term between the ex-ante control variables (2004) and the time trend is incorporated, and the regression is re-estimated accordingly. Column (5) shows that the coefficient of the policy variable is 0.224, remaining statistically significant at the 1% level, indicating that our baseline findings are robust to this alternative way of addressing potential time-varying omitted variables. In addition, Column (6) presents the results after accounting for potential confounding effects of the three grain subsidy policies, with the coefficient remaining positive and significant, further corroborating the robustness and reliability of the findings.

## 5. Mechanism Tests

Previous analysis has confirmed that the RPMGC significantly increases county-level grain production. However, the intermediate mechanisms through which the policy influences county-level grain production remain unclear. Although the funds allocated under the RPMGC function as general transfer payments and are managed at the discretion of local governments, the policy includes performance evaluation measures to maximize its stimulative effect on grain production. These measures guide the allocation of funds toward grain production, indicating that the reward funds possess a certain degree of specificity and project orientation. A review of the officially reported uses of the reward funds in several major grain-producing counties shows that the funds are primarily allocated to agricultural machinery subsidies, procurement of agricultural inputs, water conservancy maintenance, and high-standard farmland construction. Building on this, and integrating theoretical analysis with the actual use of reward funds, we further explore how the policy influences grain production, focusing on three channels: fostering technological advancement, mitigating production risks, and facilitating scaled-up production.

To investigate the underlying mechanisms through which the RPMGC affects grain production, this section adopts the approach of Shi et al. (2025) [38] by replacing the dependent variable with mechanism variables. Specifically, fostering technological advancement is proxied by agricultural mechanization level and fertilizer application amount, mitigating production risks by effective irrigated area, and facilitating scaled-up production by cultivated area per laborer. These variables are sequentially incorporated into Model (1) for regression analysis, with the results reported in Table 5. Column (1) shows that the coefficient of policy is 0.205 and statistically significant at the 1% level. Column (2) shows that the coefficient of policy is 0.538 and significant at the 1% level, indicating that the policy enhances agricultural mechanization and fertilizer application, thereby fostering technological advancement and improving the efficiency of labor and land inputs. Column (3) shows that the coefficient of policy is 0.479 and significant at the 1% level, suggesting that the policy increases effective irrigated area, which mitigates production risks by optimizing water and soil resource allocation and enhancing resilience to natural disasters. Column (4) takes cultivated area per laborer as the outcome. The estimated policy effect is positive and statistically significant, implying that the policy encourages scaled up production and thereby raises grain yield per unit area. In summary, the RPMGC fosters technological advancement, mitigates production risks, and facilitators scaled-up production, thereby increasing county-level grain production and confirming Hypothesis 2.

## 6. Heterogeneity Analysis

The preceding findings indicate that the RPMGC has significantly enhanced grain production. Further heterogeneity analyses examine whether the policy effects vary across major grain-producing regions or under different levels of fiscal pressure.

### 6.1. Heterogeneity Analysis: Regional Differences

In 2003, China’s Ministry of Finance formally classified regions into major grain-producing areas, major grain-purchasing areas, and major grain-balanced areas, based on grain output and related indicators. Major grain-producing areas are characterized by higher grain output, a long history of cultivation, and distinctive natural resource endowments. Given the significant disparities in resource endowments and policy support between these regions, the effect of the RPMGC on grain production may differ accordingly. Using this framework, the paper conducts heterogeneity analyses, with the results presented in Table 6 confirming that the effects of the policy vary significantly across the three types of regions.

The effects of the RPMGC on grain production differ markedly across major grain-producing areas, major grain-purchasing areas, and major grain-balanced areas. In major grain-purchasing and major grain-balanced areas, the policy effect is negative and statistically insignificant, whereas in major grain-producing areas the effect is positive and statistically significant at the 1% level. Several factors may account for this pattern. First, compared with major grain-purchasing and major grain-balanced areas, major grain-producing areas bear greater food security responsibilities, and their stronger grain production base provides advantages in accessing the RPMGC, thereby offering greater incentives to enhance fiscal support for agriculture and increase grain yield. Second, in major grain-purchasing and major grain-balanced areas, the grain sector typically accounts for a smaller share of the local economy, and the opportunity cost of expanding grain production, particularly in terms of foregone industrial and service-sector development, is relatively high. Under such conditions, the reward funds may be treated more as general fiscal resources and are less likely to induce the reallocation of land and other factors toward grain production, which is consistent with the statistically insignificant and marginally negative coefficients observed for these areas.

These findings suggest that when strengthening the RPMGC, priority support should be directed toward major grain-producing areas. Simultaneously, attention should be paid to potential “free-riding” behaviors by major grain-purchasing and major grain-balanced areas concerning national food security. It is essential to further clarify the food security responsibilities of each region, reconcile the interests and supply–demand relationships between production and consumption areas, and promote horizontal benefit compensation across these regions.

### 6.2. Heterogeneity Analysis: Financial Pressures

County-level governments in China shoulder multiple fiscal responsibilities: In counties with limited financial capacity, resource constraints are common, and transfers from higher-level governments constitute a crucial source of funding for wages, operational costs, and social welfare expenditures. Therefore, relative to counties under lower fiscal pressure, those experiencing higher fiscal pressure demonstrate a stronger and more urgent demand for the RPMGC, potentially resulting in increased investment in grain production to obtain the incentives. Local fiscal pressure is measured as the ratio of the fiscal revenue–expenditure gap to total fiscal revenue [25], and the sample is divided into two groups according to the median fiscal pressure for regression analysis. The coefficient of policy is 0.477 for the high fiscal pressure group and 0.306 for the low fiscal pressure group, both statistically significant at the 1% level, indicating that the policy promotes grain production regardless of fiscal pressure. However, coefficient comparisons indicate a larger policy effect in counties under greater fiscal pressure. In summary, the RPMGC should be further refined and strengthened, with additional support targeted at counties experiencing higher fiscal pressure. Simultaneously, efforts should aim to improve the provision of basic public services in major grain-producing counties, gradually reduce fiscal pressure, and foster a positive incentive mechanism that encourages local governments to prioritize agriculture, emphasize grain production, and reward higher output.

## 7. Conclusions and Discussion

Using the RPMGC as an entry point, this research leverages a county-level panel of 1319 counties spanning 2000–2021 to systematically evaluate the incentive effects of fiscal transfers on grain production. The study quantifies the policy’s overall effect and further examines how the effect varies over time, through which channels it operates, and the heterogeneity in policy responses, thereby offering an integrated assessment of how fiscal incentives contribute to national food security capacity.

First, our estimates indicate that RPMGC significantly increases grain yield per unit area. This finding is robust across multiple sensitivity analyses, including tests for parallel trends, placebo tests, instrumental variable estimation, and adjustments to sample size and control variables. Against the backdrop of tightly constrained arable land resources, fiscal transfers have played a substantive role in strengthening grain production capacity and are directly relevant to food security objectives. Meanwhile, we find that the policy effect is dynamic, exhibiting a “rise-and-fall” trajectory over time. The evidence in this paper does not suggest that the policy has “failed” in any absolute sense; rather, it reveals the time-limited nature of the incentive effect. The pronounced early effect is closely associated with the introduction of additional rewards for super-major grain-producing counties and the implementation of performance evaluation mechanisms, whereas the subsequent attenuation coincides with a slowdown in the growth rate of reward funds. Accordingly, policy design should place greater emphasis on the sustainability of incentives and the issue of diminishing marginal returns. Within fiscally affordable limits, the real incentive strength of reward funds should be moderately increased and stabilized, and a dynamic adjustment mechanism should be established that aligns with counties’ contributions to grain output growth, so as to sustain major grain-producing counties’ motivation to produce grain.

Second, mechanism analyses indicate that the policy increases grain production primarily by fostering technological advancement, mitigating production risks, and facilitating scaled-up production. Policy design and rules governing the use of funds should be further refined to steer local governments to prioritize allocating resources to key areas, including agricultural technology innovation and diffusion, risk-management investments such as farmland water conservancy and disaster prevention and control, and support for appropriately scaled-up production. This would help prevent overly generalized use of funds that could dilute support for the core mechanisms driving grain output growth.

Finally, the heterogeneity analysis shows that RPMGC delivers stronger grain output gains in major grain-producing areas and in areas under greater fiscal pressure; in contrast, in major grain-purchasing areas and major grain-balanced areas, as well as in areas facing lower fiscal pressure, it is difficult to achieve comparable incentive effects. This indicates that the effectiveness of fiscal transfers depends to a large extent on the alignment between food security responsibilities, resource endowments, and fiscal capacity. Accordingly, future support should be strategically tilted toward core grain-producing areas and counties confronting greater fiscal pressure. Implementing such a differentiated support strategy would enhance overall allocative efficiency and ensure that limited resources yield the largest possible incentive effects.

Overall, this study makes three principal contributions: First, it provides empirical evidence supporting the effectiveness of fiscal transfers in promoting grain production. Second, it extends the empirical literature by identifying specific mechanisms through which fiscal incentives affect grain output. Third, it reveals the context-dependent nature of policy effectiveness across regional types and varying levels of fiscal pressure, thereby providing robust empirical foundations for optimizing the design of incentive-based fiscal transfers.

However, this study has certain limitations. Because county-specific incentive amounts are not publicly disclosed, we are unable to identify how funds are allocated across uses within counties, let alone evaluate their effects at the household level. Relatedly, given these data constraints, our mechanism tests rely mainly on indirect evidence. Future research could combine case studies and field interviews with fiscal execution ledgers or household level microdata to trace fund flows and assess microlevel efficiency.

## Figures and Tables

**Table 1:** Definition of main variables and descriptive statistics.

Variable	Definition	Mean	SD
Yield	Grain yield per unit area	5.045	1.674
Policy	=1 if a county received the reward for major grain-producing counties in year t and afterwards; =0 otherwise	0.292	0.455
Primary industry	Value added of primary industry/GDP	24.591	13.159
Secondary industry	Value added of secondary industry/GDP	39.129	15.722
Financial	The balance of loans from financial institutions/GDP	59.899	34.675
Household saving	Personal savings deposits/GDP	70.562	34.746
Government	Financial expenditure/GDP	24.671	21.988
Regional area	Administrative land area of the region	4002.976	10,276.3
Temperature	Annual average temperature	14.097	5.218
Precipitation	Annual precipitation	1042.667	545.057
Crop-sown area	Total sown area of crops in the region	7.078	6.230
Agricultural mechanization level	Total power of agricultural machinery	0.360	0.377
Fertilizer application amount	Fertilizer application amount calculated on a pure nutrient basis	2.364	2.647
Effective irrigated area	Effective irrigated area	2.599	2.845
Cultivated area per laborer	Grain-sown area per rural laborer	0.154	0.139

**Table 2:** The regression estimates of the benchmark analysis.

Variables	Yield	
	(1)	(2)
Policy	0.298 ***	0.316 ***
	(0.046)	(0.043)
Control variables	No	Yes
Year fixed effect	Yes	Yes
County fixed effect	Yes	Yes
*R* ^2^	0.820	0.824
*N*	29,018	29,018

Note: *** denotes significance at 1% and the robust standard errors are reported in parentheses, clustered by county.

**Table 3:** Results of the instrumental variables approach.

Variables	Phase I	Phase II
	Policy	Yield
Policy		0.259 *
		(0.145)
IV	−0.084 ***	
	(0.005)	
Control variables	Yes	Yes
Year fixed effect	Yes	Yes
County fixed effect	Yes	Yes
*N*	29,018	29,018
Kleibergen–Paap rk LM	243.655 [0.000]	
Kleibergen–Paap rk Wald F	303.537 {16.38}	

Note: *** and * denote significance at the 1% and 10% levels, respectively. Values in () are robust standard errors; values in [] are *p*-values; values in {} are the 10% critical values from the Stock–Yogo weak identification test.

**Table 4:** Robustness test results.

Variables	Yield					
	Substitution of Explained Variable		Adjustment of Sample Size		Adjustment of Control Variables	Exclusion of Other Policies
	(1)	(2)	(3)	(4)	(5)	(6)
Policy	0.231 ***	0.206 ***	0.308 ***	0.356 ***	0.224 ***	0.296 ***
	(0.017)	(0.014)	(0.044)	(0.040)	(0.047)	(0.042)
Control variables	Yes	Yes	Yes	Yes	Yes	Yes
Year fixed effect	Yes	Yes	Yes	Yes	Yes	Yes
County fixed effect	Yes	Yes	Yes	Yes	Yes	Yes
*R* ^2^	0.959	0.964	0.824	0.853	0.824	0.824
*N*	29,017	29,018	27,699	19,785	29,018	29,018

Note: *** denotes significance at 1%, and the robust standard errors are reported in parentheses, clustered by county. The control variables in Column (4) of Table 4 are the interaction terms of the ex-ante control variables (2004) and the time trend, while the control variables in the other columns are time-varying control variables.

**Table 5:** Mechanism test results.

Variables	Agricultural Mechanization	Fertilizer Application	Effective Irrigated Area	Cultivated Area per Laborer
	(1)	(2)	(3)	(4)
Policy	0.205 ***	0.538 ***	0.479 ***	0.046 ***
	(0.009)	(0.071)	(0.070)	(0.003)
Control variables	Yes	Yes	Yes	Yes
Year fixed effect	Yes	Yes	Yes	Yes
County fixed effect	Yes	Yes	Yes	Yes
*R* ^2^	0.913	0.858	0.797	0.886
*N*	29,018	29,018	29,018	29,001

Note: *** denotes significance at 1%, and the robust standard errors are reported in parentheses, clustered by county.

**Table 6:** Heterogeneity test results.

Variables	Yield				
	Major Grain-Producing Areas	Major Grain-Purchasing Areas	Major Grain-Balanced Areas	High Financial Pressures	Low Financial Pressures
	(1)	(2)	(3)	(4)	(5)
Policy	0.370 ***	−0.215	−0.049	0.477 ***	0.306 ***
	(0.054)	(0.208)	(0.071)	(0.079)	(0.053)
Control variables	Yes	Yes	Yes	Yes	Yes
Year fixed effect	Yes	Yes	Yes	Yes	Yes
County fixed effect	Yes	Yes	Yes	Yes	Yes
*R* ^2^	0.816	0.721	0.819	0.820	0.779
*N*	16,060	2266	8690	15,224	13,794

Note: *** denotes significance at 1%, and the robust standard errors are reported in parentheses, clustered by county.

## Data Availability

The data that support the findings of this study are available from the corresponding author upon reasonable request.
